# Astrogliosis in the GFAP-Cre^ERT2^:Rosa26^iDTR^ Mouse Model Does Not Exacerbate Retinal Microglia Activation or Müller Cell Gliosis under Hypoxic Conditions

**DOI:** 10.3390/biom14050567

**Published:** 2024-05-10

**Authors:** Colin Rorex, Sandra M. Cardona, Kaira A. Church, Derek Rodriguez, Difernando Vanegas, Reina Saldivar, Brianna Faz, Astrid E. Cardona

**Affiliations:** 1 Molecular Microbiology and Immunology, College of Sciences, The University of Texas at San Antonio, San Antonio, TX 78249, USA; colin.rorex@utsa.edu (C.R.);; 2 Integrative Biology, The University of Texas at San Antonio, San Antonio, TX 78249, USA; 3 South Texas Center for Emerging Infectious Diseases, The University of Texas at San Antonio, San Antonio, TX 78249, USA

**Keywords:** diabetic retinopathy, microglia, astrocytes, Müller glia, hypoxia, inflammation, fibrinogen

## Abstract

Diabetic retinopathy (DR) affects over 140 million people globally. The mechanisms that lead to blindness are still enigmatic but there is evidence that sustained inflammation and hypoxia contribute to vascular damage. Despite efforts to understand the role of inflammation and microglia in DR’s pathology, the contribution of astrocytes to hypoxic responses is less clear. To investigate the role of astrocytes in hypoxia-induced retinopathy, we utilized a 7-day systemic hypoxia model using the GFAP-Cre^ERT2^:Rosa26^iDTR^ transgenic mouse line. This allows for the induction of inflammatory reactive astrogliosis following tamoxifen and diphtheria toxin administration. We hypothesize that DTx-induced astrogliosis is neuroprotective during hypoxia-induced retinopathy. Glial, neuronal, and vascular responses were quantified using immunostaining, with antibodies against GFAP, vimentin, IBA-1, NeuN, fibrinogen, and CD31. Cytokine responses were measured in both the brain and serum. We report that while both DTx and hypoxia induced a phenotype of reduced microglia morphological activation, DTx, but not hypoxia, induced an increase in the Müller glia marker vimentin. We did not observe that the combination of DTx and hypoxic treatments exacerbated the signs of reactive glial cells, nor did we observe a significant change in the expression immunomodulatory mediators IL-1β, IL2, IL-4, IL-5, IL-6, IL-10, IL-18, CCL17, TGF-β1, GM-CSF, TNF-α, and IFN-γ. Overall, our results suggest that, in this hypoxia model, reactive astrogliosis does not alter the inflammatory responses or cause vascular damage in the retina.

## 1. Introduction

The deterioration of retinal microcirculation due to vascular damage can lead to hypoxic responses. As a result, an increase in the mechanisms used to mobilize the number of blood cells to transport oxygen, including hematopoiesis and the number of blood vessels (angiogenesis), is observed [1]. Hypoxia is involved in the pathological process of inflammatory neurodegenerative diseases such as Alzheimer’s disease, Parkinson’s disease, and amyotrophic lateral sclerosis, and retinal diseases such as diabetic retinopathy (DR), retinal vein occlusion, age-related macular degeneration, and glaucoma [2,3]. In DR patients, signs of retinal hypoxia are evident in their increased levels of hypoxia inducible factor-1α (HIF-1α), vascular endothelial growth factor (VEGF), and erythropoietin (EPO). Changes in the expression of these factors are correlated with disease and a progression from non-proliferative to proliferative DR [4,5]. Additionally, electroretinography from DR patients shows a decrease in the amplitude of their oscillatory potentials similar to those reported in individuals undergoing hypoxic environmental stress [4,6].

Microglia, the resident phagocyte of CNS tissues, are a principal source of inflammatory mediators in the retina. In DR pathology, microglia respond to multiple signals of dysregulated homeostasis with an inflammatory response [7]. The metabolic byproducts of sustained hyperglycemia, advanced glycated end products (AGEs), induce inflammatory responses via the AGE receptor (RAGE), inducing an NF-κB response [8]. Hypoxia induces an inflammatory microglial response via HIF-1α and STAT1 transcription factors, leading to the secretion of IL-1β, IL-6, and TNF-α [9,10]. Fibrinogen accumulation in affected tissues due to damaged endothelial cells also contributes to tissue damage by sustaining an inflammatory response that can be rescued by deleting the c-terminal domain of fibrinogen, preventing its interaction with microglial CD11b [11,12].

Hypoxic or oxidative stress induce responses in other resident CNS populations including neurons, astrocytes, and endothelial cells. For example, hypoxic neurons secrete molecules such as prokineticin-2 [13]. In contrast to the hypoxic response in microglia, the activation of the prokineticin-2 receptor on astrocytes induces an anti-inflammatory response with an upregulation of STAT6, reducing the expression of pro-inflammatory cytokines IL-1β, IL-6, and TNF-α, and and increased expression of the glutamate-aspartate transporter, enhancing astrocyte uptake and the recycling of glutamate [13,14]. The activation of HIF-1α induces a signaling cascade through miR-101 that downregulates VE-cadherin and claudin-5, decreasing endothelial integrity and increasing vascular leakage [15]. As demonstrated in a mouse model of systemic hypoxia, this degradation of vascular integrity manifests as an accumulation of both intra-vascular and extra-vascular fibrinogen in brain tissue [16].

In DR, astrocytes are affected by sustained inflammation and hypoxia signals that induce opposing neuroprotective and neurotoxic responses [17]. In our previous work, we investigated the role of FKN and CX3CR1 in the pathological mechanism of DR [18,19,20,21,22]. Microglial neurotoxic responses are regulated by neurons, in part via the production of CX3CL1 (fractalkine, FKN), a chemokine that binds to the CX3CR1 expressed on microglia [18]. The disruption of the FKN-CX3CR1 signaling axis increased microglia reactivity and inflammatory responses in several neuroinflammatory diseases [23]. We found that disrupting the FKN-CX3CR1 signal axis detrimentally affected neuronal health and worsened disease severity and visual function in mouse models of DR. In *Cx3cr1^–/–^* mice, whose microglia showed enhanced inflammatory responses, we found evidence of IL-1β expression by GFAP^+^ astrocytes with a corresponding decrease in their overall GFAP immunoreactivity area at 20 weeks of diabetes [19]. In the hyperglycemic *Cx3cl1^–/–^* model we also observed, as in the *Cx3cr1^–/–^* mice, increased inflammation and GFAP^+^ immunoreactivity, and decreased visual function that was rescued by FKN gene therapy [22]. Microglia depletion and replenishment during streptozotocin (STZ)-induced hyperglycemia in wild-type mice resulted in an increase in homeostatic microglia, with ramified morphology, that was associated with increases in axonal health and GFAP^+^ immunoreactivity [21]. These data suggest that aberrant microglia activation is associated with increased inflammatory astrocyte responses and an exacerbation of the inflammatory pathology in the retina. To extend those studies, we seek to understand astrocyte responses during hypoxia and whether the hypoxic response can be modulated by an in vivo induction of reactive astrogliosis.

To assess the role of reactive astrocytes in regulating tissue pathology, we utilized a model of systemic hypoxia, in which GFAP-Cre^ERT2^:Rosa26^iDTR^ adult mice were exposed to 7.5% O_2_. In this genetic model system, a Cre-fusion protein is expressed under the GFAP promoter that is responsive to tamoxifen administration, enabling the conditional expression of a diphtheria toxin receptor (DTR) and, therefore, astrocyte-specific diphtheria toxin (DTx) sensitivity. The DTR-DTx system has been highly successful in exerting short-term temporal control of particular cell types [24]. However, in the brain, DTx-mediated depletion protocols showed a reactive inflammatory astrocyte response instead of the depletion of GFAP+ astrocytes [25]. Therefore, we hypothesize that DTx-induced astrogliosis will induce an anti-inflammatory response, ameliorating the inflammatory pathology of hypoxia-induced retinopathy with a decrease in the reactive microglia, vascular pathology, and increased expression of anti-inflammatory mediators. We found that DTx-mediated reactive astrogliosis also induced signs of Müller glia activation. While both DTx and hypoxic treatments induce a reduction in the ramification of microglia, the combination of DTx and hypoxia did not exacerbate microglial morphological activation. We did not observe evidence of vascular pathology in response to hypoxia, DTx administration, or a combination of the two. Lastly, we observed no change in the expression of pro-inflammatory and anti-inflammatory mediators that correlate to the observed tissue pathology. Overall, this suggests that hypoxic signals from reactive astrocytes do not exacerbate the inflammatory processes in retinal tissue.

## 2. Materials and Methods

### 2.1. Animals

**Figure 1:**
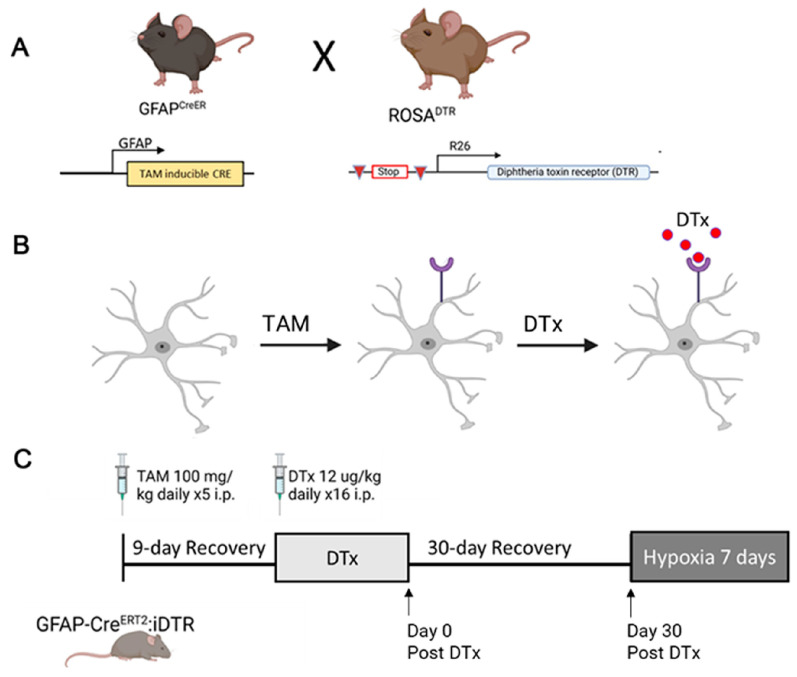
Experimental design. (**A**) Breeding scheme to generate the GFAP-Cre^ERT2^:Rosa26^iDTR^ mouse line. (**B**) Administration of TAM induces the translocation of the Cre^ERT2^ protein, removing the upstream stop codon from the Rosa26iDTR gene, inducing the expression of a variant DTR, resulting in sensitivity to the DTx toxin in astrocytes. (**C**) Timeline of treatments for all experiments. Arrows indicate tissue collection time points for all experiments, with Day 0 post DTx, Day 30 post DTx, and hypoxic groups.

The GFAP-Cre^ERT2^:Rosa26^iDTR^ mouse line was generated by crossing the B6.Cg-Tg (GFAP-cre/ERT2)505Fmv/J (Jackson Laboratories, Bar Harbor, ME, USA, strain #012849) mouse with the Rosa26:iDTR (Jackson Laboratories, Bar Harbor, ME, USA, strain #007900) mouse (Figure 1). Males and females hemizygous for both alleles were utilized in all experiments. Animals were 6–12 weeks of age at the start of all experiments.

### 2.2. Diphtheria Toxin-Mediated Astrocyte Activation in the GFAP-Cre^ERT2^:Rosa26^iDTR^ Mouse

Induction of Cre recombination: mice at least 6 weeks of age were injected with 100 mg/kg tamoxifen (TAM) (Sigma Aldrich, St. Louis, MO, USA, #T5648) daily for 5 days via I.P. injection (Figure 1B,C), and then allowed to recover for a 9-day period. Diphtheria toxin (DTx) administration: following the TAM recovery period, mice were injected with either a PBS vehicle control or 12 ng DTx/g (Sigma Aldrich, St. Louis, MO, USA, #D0564) of their body weight daily for 16 days (Figure 1C). To quantify the astrocyte responses to DTx, retina, brain, and peripheral blood were collected on the day of the last DTx injection (Day 0 post-DTx), Day 30 post DTx administration, or after 7 days of hypoxic conditions (Figure 1C).

### 2.3. Induction of Hypoxia-Induced Retinopathy

Mice were exposed to hypoxic conditions of 7.5% oxygen for 7 days in an airtight chamber (Coy Laboratory Products, Grass Lake, MI, USA, InVivo Cabinet model 15) in open-air housing cages with free access to food and water. Oxygen levels were maintained by the injection of supplemental N_2_ and O_2_ gas. Animals were monitored twice daily and returned to normoxic conditions on an as-needed basis for periods no longer than 30 min for injections or due to welfare concerns. Age-matched control animals were maintained under normoxic conditions (~20.6% O_2_, the ambient oxygen levels of San Antonio Texas).

### 2.4. Tissue Collection

Anesthetized mice were transcardially perfused with cold 1× Hanks’s Balanced Salt Solution (HBSS, Fisher Scientific, Pittsburgh, PA, USA, BW10-547F). Enucleated whole globes were fixed in 4% paraformaldehyde (Sigma Aldrich, St. Louis, MO, USA, #P6148, PFA) for 20 min. Retinas were then dissected and transferred, along with optic nerves, to 1% PFA. Brains collected for histology were fixed overnight in 4% PFA. Fixed tissues were then prepared for long-term storage as previously described [20]. Briefly, fixed tissues were cryoprotected (200 mL glycerol, 200 mL 0.4 M Sorenson’s buffer, and 600 mL water) overnight at 4 °C then transferred to a cryostorage solution (500 mL 0.2 M PO4, 10 g PVP-40, 300 g sucrose and 300 mL ethylene glycol) and stored at −20 °C. Brain protein extracts were collected by mechanical homogenization in protein extraction buffer (9.15 mL water, 0.6 mL 2.5 M NaCl, 0.1 mL 1 M Tris base, 20 μL 0.5 M EDTA, and 100 μL of protease inhibitor cocktail (Millipore Sigma, Burlington, MA, USA, #0469311600)) and the protein supernatant was collected by centrifugation at 4 °C and 12,000 RPM, for 15 min then stored at −80 °C. Peripheral blood was collected by a puncture of the submandibular vein, and blood (200–400 μL) was collected in EDTA-treated tubes (BD, Franklin Lakes, NJ, USA, #365974). Blood was centrifuged for 20 min at 2000× *g* and 4 °C. Plasma was transferred to sterile 0.6 mL tubes with 1 μL of protease inhibitor cocktail per 100 μL of plasma, then stored at −80 °C.

### 2.5. Immunofluorescent Staining

Tissues were prepared utilizing protocols that have been described previously [20]. In brief, retinas were cut into 4 radial pieces and selected at random to be stained for the markers of interest (Table 1). Tissues were blocked overnight in blocking solution (450 μL 10% normal goat serum, 450 μL 10% normal donkey serum, and 100 μL of 10% Triton/mL) at 4 °C. Then, tissues were incubated overnight at 4 °C with primary antibodies (Table 1) diluted in fresh blocking solution. Unbound primary antibodies were removed by 5 washes, of 5 min each, in PBS with 0.1% Triton at room temperature. The visualization of primary antibodies was achieved by their incubation with host-specific secondary antibodies for 3 h at room temperature (Table 1); indirect staining of vimentin utilized a biotinylated anti-chicken secondary antibody subsequently visualized using streptavidin-conjugated Cy3. Tissues were washed as described previously, and then their nuclei were stained with Hoechst 3342 (Thermo Fisher Scientific, Pittsburgh, PA, USA, #H1399) diluted 1:1000 in PBS for 7 min at room temperature. Unbound Hoechst was removed from tissues by washing 5 times in PBS before the tissues were mounted on superfrost plus microscope slides (Fisher Scientific, Pittsburgh, PA, USA, #12-550-15) in Fluorsave reagent (Millipore Sigma, Burlington, MA, USA, #345789).

### 2.6. Tissue Imaging and Quantification

Confocal images were obtained at 40× magnification using a Zeiss 710 NLO 2P confocal microscope at the UTSA Cell Analysis Core (University of Texas at San Antonio, San Antonio, TX, USA). Six images were obtained per retinal leaflet, with 3 from the central and 3 from the peripheral region of the retina. Image analysis and processing was performed utilizing IMARIS (Oxford Instruments, Abingdon, Oxfordshire, UK, version 6.4), Photoshop (Adobe, San Jose, CA, USA, version 22.1), and Fiji (version 1.53f53) [26] software. Cell densities were quantified using the object counter tool in Photoshop and Fiji. All quantifications were performed by two readers blinded to experimental cohort metadata. The immunoreactive area was calculated by converting images in ImageJ to 32-bit grayscale images and setting manual thresholds to match raw image intensity. Astrocyte (S100β^+^ cell bodies co-localizing to GFAP^+^ processes) and retinal ganglion cell (RGC, NeuN^+^ cell bodies) densities were calculated by normalizing the cell counts to volume based on the Z-axis depth within each tissue. Microglia reactivity was calculated using the transformation index, as described previously [27]. In brief, microglia were traced using Fiji to determine their perimeter and area, and then their TI was calculated using the following equation: perimeter^2^/(4π × area^2^).

### 2.7. Protein Quantification

Brain protein and serum samples were assayed for their cytokine expression levels utilizing multiplexed ELISA technologies. The Bio-plex platform was utilized to quantify IL-1β, IL-2, IL-4, IL-5, IL-6, IL-10, IFN-γ, TNF-α, and GM-CSF in a custom Mouse Cytokine panel on a Bio-Plex 200 (Bio-Rad, Hercules, CA, USA, #17007670). CCL17 and IL-18 were quantified with the LEGENDplex^®^ Mouse Macrophage/Microglia Panel Mix and Match kit (BioLegend, San Diego, CA, USA, #740855 & 740864). LEGENDplex^®^ samples were analyzed on a FACS Celesta (BD Biosciences, San Jose, CA, USA) with a high-throughput sampler. Assays were performed per the manufacturer’s instructions and system settings. Samples were normalized to volume, for serum samples, or to total protein, for brain lysates.

### 2.8. Statistical Analysis

Our statistical analyses utilized GraphPad Prism v9.5.1 and the statistical significance indicators are denoted as follows: * *p* < 0.05, ** *p* < 0.01, *** *p* < 0.001, and **** *p* < 0.0001. Comparisons between two data elements utilized a two-tailed non-parametric Mann–Whitney *T*-test (Supplementary Table S1). A Power Analysis estimated the cohort sizes using the following equation: N = 2$\sigma {\left(\frac{Z\alpha -Z\beta }{\mu 1-\mu 2}\right)}^{2}$. Zα, assuming a two-sided test and an α of 0.05, =1.960 and Zβ, assuming a power of 0.95, =1.645; the mean assay levels μ1 and μ2 were 100 and 95, respectively. Thus, we estimated cohort sizes of N = 5–10 for mean comparisons.

## 3. Results

### 3.1. Diphtheria Toxin Administration in GFAP-Cre^ERT2^:Rosa26^iDTR^ Mice Is Associated with Reactive Astrogliosis and Vimentin Overexpression

**Figure 2:**
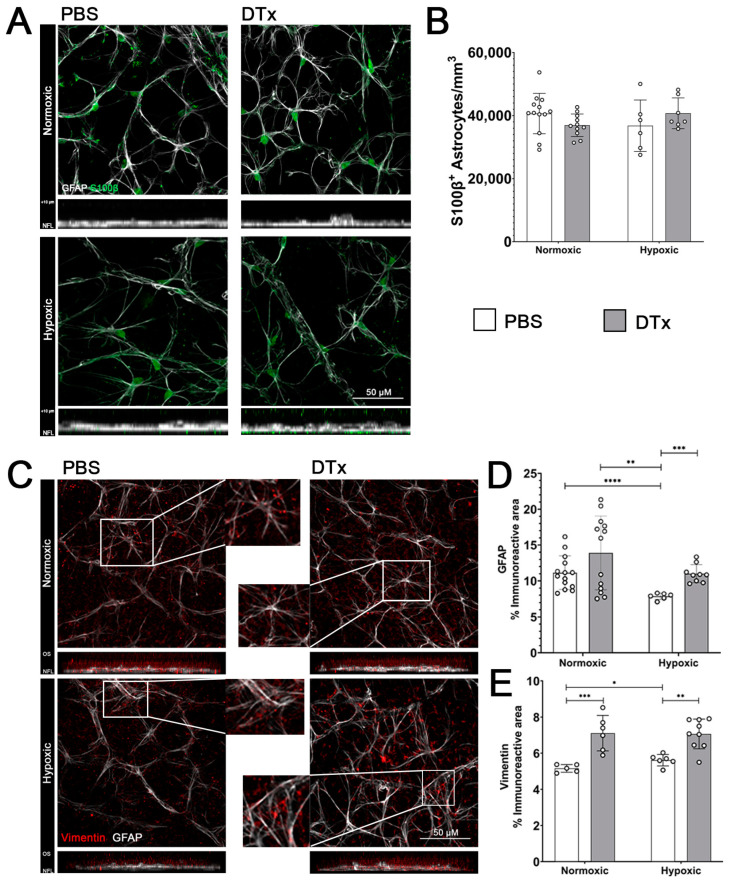
DTx induces an increase in the GFAP-immunoreactive area in DTx-treated hypoxic retinas, while vimentin expression is increased independent of a hypoxic treatment. (**A**) Representative 40× magnification confocal images of mouse retinas stained with astrocyte marker GFAP (white) and S100β (green), with transverse rotation (inset). (**B**) Quantification of astrocyte densities of the immunoreactive area for GFAP and vimentin, respectively. (**C**) Representative 40× magnification confocal images of mouse retinas stained with astrocyte marker GFAP (white) and Müller glia marker vimentin (red), with transverse rotation (inset). (**D**,**E**) Quantification of GFAP- and vimentin-immunoreactive areas, respectively. Data are presented as mean ± SD, n = 5–13 mice per group, dots indicate averages for individual mice. Scale bar measures 50 μm. * *p* < 0.05, ** *p* < 0.01, *** *p* < 0.001, and **** *p* < 0.0001.

To elucidate the role of astrogliosis in hypoxia-induced retinal pathology, we utilized the inducible DTR-DTx model to manipulate astrocytes in vivo. We hypothesized that the retinal astrocytes in the GFAP-Cre^ERT2^:Rosa26^iDTR^ mouse would respond with the same phenotype of delayed reactivity and no evidence of depletion, a response similar to that which has been reported in the brain [19]. We tested the effects of hypoxic conditions and DTx administration on astrocyte density by staining for GFAP (white) and calcium binding protein S100β (green) (Figure 2A). This double stain allows individual astrocytes to be distinguished from the GFAP network. Astrocyte density was calculated by quantifying cS100β+ astrocytes and normalized to the average volume of the RGC layer (Figure 2B). Hypoxia did not affect astrocyte density (Figure 2A,B), and normoxic tissues showed (40,660 ± 6412 astrocyte/mm^3^) similar cellular densities to hypoxic PBS retinas (36,797 ± 8155 astrocytes/mm^3^, Mann–Whitney *T*-test *p* = 0.2026). Similarly, hypoxia did not affect the astrocyte density in DTx-treated tissues (DTx normoxic 36,941 ± 8155 astrocytes/mm^3^, DTx hypoxic 40,737 ± 4894, Mann–Whitney *T*-test *p* = 0.1200). These data show that DTx did not affect astrocyte proliferation or depletion under our tested conditions.

Next, we assessed how hypoxia and DTx alter astrocytes’ reactivity by quantifying changes in GFAP expression (Figure 2C,D). We observed that, under normoxic conditions, DTx-treated mice show an astrocyte phenotype that resembles those of PBS normoxic control astrocytes (PBS normoxic 11.142 ± 2.346%, DTx normoxic 13.908 ± 5.145%, Mann–Whitney *T*-test *p* = 0.3255). However, PBS control retinas under hypoxic conditions showed a significant decrease in GFAP immunoreactivity compared to normoxic retinas (PBS hypoxic 7.852 ± 0.439%, vs. PBS normoxic Mann–Whitney *T*-test *p* = 0.0001, vs. DTx normoxic Mann–Whitney *T*-test *p* = 0.0066). The combination of DTx and hypoxia induced an increase in GFAP immunoreactivity compared to hypoxic PBS tissues (DTx hypoxic 11.011 ± 1.265%, vs. PBS hypoxic Mann–Whitney *T*-test *p* = 0.0004), but not compared to PBS or DTx normoxic tissues (DTx hypoxic vs. PBS normoxic Mann–Whitney *T*-test *p* = 0.9263, DTx hypoxic vs. DTx normoxic Mann–Whitney *T*-test *p* = 0.5556) (Figure 2D).

We also quantified vimentin immunoreactivity (red), a marker for Müller glia [28] (Figure 2C,E). We observed a significant increase in vimentin immunoreactivity in DTx-treated retinas (PBS normoxic 5.225 ± 0.179%, DTx normoxic 7.109 ± 0.98%, Mann–Whitney *T*-test *p* = 0.0043), with a similar response in hypoxic retinas (PBS hypoxic 5.512 ± 0.398%, DTx hypoxic 7.066 ± 0.821%, Mann–Whitney *T*-test *p* = 0.0074). These data indicate that DTx induced an increase in GFAP immunoreactivity and that DTx-induced Müller glial activation occurs independent of hypoxic conditions.

### 3.2. Reactive Astrogliosis in the GFAP-Cre^ERT2^:Rosa26^iDTR^ Mouse Does Not Affect Vascular Integrity

**Figure 3:**
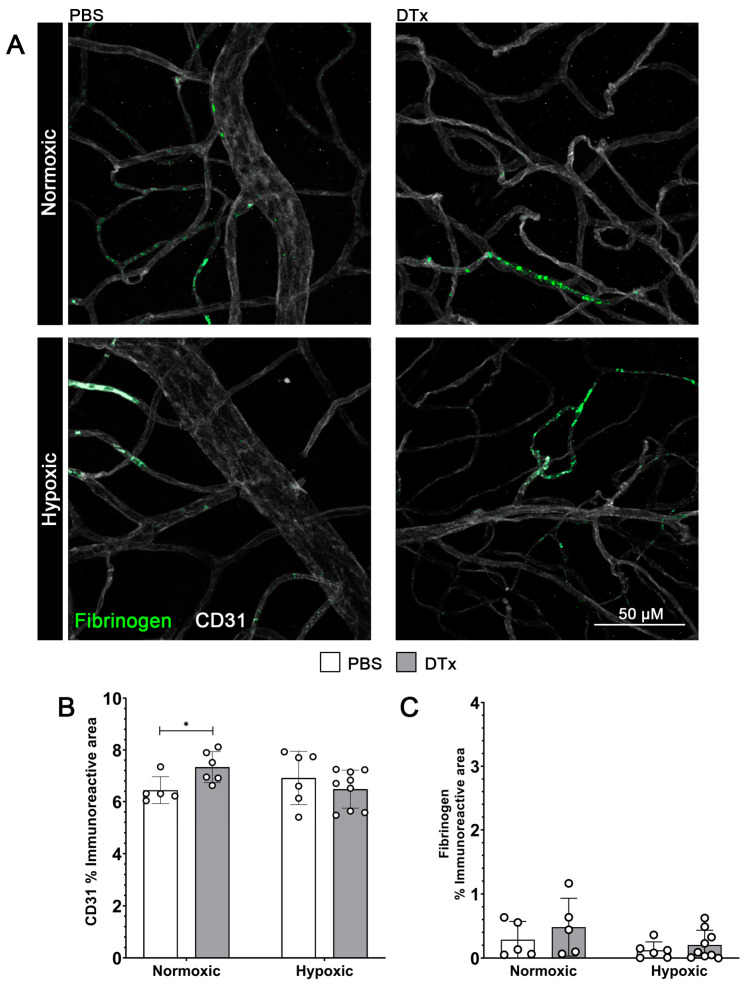
Hypoxic GFAP-Cre^ERT2^:Rosa26^iDTR^ mouse retinas show no evidence of vascular pathology. (**A**) Representative 40× magnification confocal images of mouse retinas stained with endothelial cell marker CD31 (white) and fibrinogen (green). (**B**,**C**) Quantification of immunoreactive area of CD31 and fibrinogen, respectively. Data were analyzed for statistical significance between PBS- and DTx-treated animals under normoxic and hypoxic conditions. Data are presented as mean ± SD, n = 5–9 mice per group, and dots indicate averages for individual mice. Scale bar measures 50 μm. * *p* < 0.05.

Both astrocytes and Müller glia directly interact with retinal vasculature and are involved in processes such as VEGF signaling, which is associated with angiogenic responses [29]. We next tested the hypothesis that increased macroglia reactivity would correlate to increased vascular pathology. Here we quantified vascular damage by quantifying the extravasation of the blood clotting factor fibrinogen (green) into retinal tissues (Figure 3A) [12]. To quantify the changes in retinal vascular structures, tissues were stained with CD31 (Figure 3A). While we did observe an increase in the CD31 immunoreactive area in normoxic control tissues in response to DTx, we did not observe significant differences between the other conditions (PBS normoxic 4.582 ± 1.06%, PBS hypoxic 5.42 ± 2.101%, DTx normoxic 7.289 ± 2.29%, DTx hypoxic 6.486 ± 0.645%, PBS normoxic vs. PBS hypoxic Mann–Whitney *T*-test *p* = 0.4286, DTx normoxic vs. DTx hypoxic Mann–Whitney *T*-test *p* = 0.0663, PBS normoxic vs. DTx normoxic Mann–Whitney *T*-test *p* = 0.0303, PBS hypoxic vs. DTx hypoxic Mann–Whitney *T*-test *p* = 0.4559) (Figure 3B). We observed no increase in fibrinogen accumulation in response to either hypoxic conditions or DTx administration (PBS normoxic 0.36 ± 0.0247%, PBS hypoxic 0.174 ± 0.153%, DTx normoxic 0.439 ± 0.152%, DTx hypoxic 0.125 ± 0.364%, PBS normoxic vs. PBS hypoxic Mann–Whitney *T*-test *p* = 0.4286, DTx normoxic vs. DTx hypoxic Mann–Whitney *T*-test *p* = 0.1469, PBS normoxic vs. DTx normoxic Mann–Whitney *T*-test *p* = 0.9307, PBS hypoxic vs. DTx hypoxic Mann–Whitney *T*-test *p* = 0.3636) (Figure 3C). These results suggest that neither DTx, hypoxia, nor the combination of the two induce vascular damage in retinal tissue.

### 3.3. Hypoxia Does Not Alter Neuronal Densities in the GFAP-Cre^ERT2^:Rosa26^iDTR^ Mouse Model

**Figure 4:**
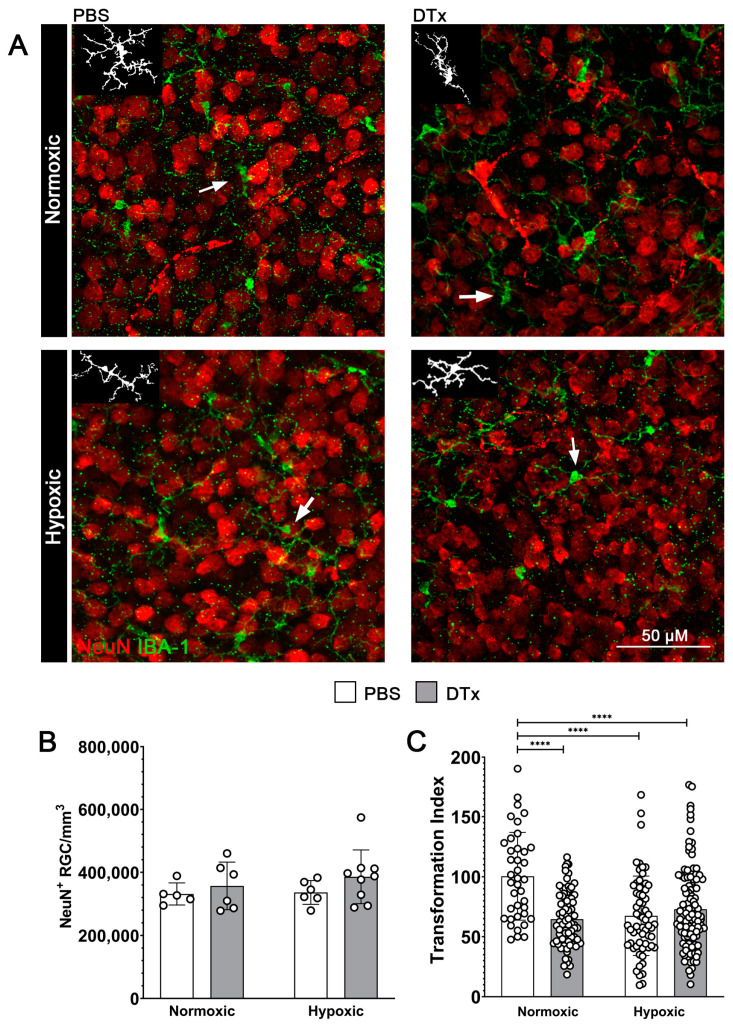
Hypoxic GFAP-Cre^ERT2^:Rosa26^iDTR^ mouse retinas do not show evidence of neurodegeneration or exacerbated microglia activation under hypoxic conditions. (**A**) Representative 40× magnification confocal images of mouse retinas stained with RGC cell marker NeuN (red) and microglial marker IBA-1 (green), with representative microglia tracings indicated by arrows (inset). (**B**) Quantification of RGC density. (**C**) Quantification of microglia morphology using the transformation index. Data were analyzed for statistical significance between PBS- and DTx-treated animals under normoxic and hypoxic conditions. Data are presented as mean ± SD, n = 5–9 mice per group, and dots indicate (**B**) averages for individual mice and (**C**) individual microglia. Scale bar measures 50 μm. **** *p* < 0.0001.

We next assessed whether the combination of hypoxia and DTx would exacerbate the inflammatory response in tissues, which in turn could induce neuronal loss [30]. Retinal tissues were stained with NeuN (red) to identify all retinal ganglion cells (Figure 4A,B). We did not observe changes in RGC densities in response to hypoxia or DTx administrations (PBS normoxic 331,430 ± 34,955 cells/mm^3^, PBS hypoxic 336,187 ± 37,872 cells/mm^3^, DTx normoxic 357,010 ± 75,590 cells/mm^3^, DTx hypoxic 385,909 ± 85,276 cells/mm^3^, PBS normoxic vs. PBS hypoxic Mann–Whitney *T*-test *p* = 0.9307, DTx normoxic vs. DTx hypoxic Mann–Whitney *T*-test *p* = 0.6889, PBS normoxic vs. DTx normoxic Mann–Whitney *T*-test *p* = 0.9307, PBS hypoxic vs. DTx hypoxic Mann–Whitney *T*-test *p* = 0.1447). These data indicate that a 7-day hypoxic treatment, in adult mice is not sufficient to induce neuronal loss.

### 3.4. Retinal Microglial Cells in DTx-Treated Mice Show Evidence of Morphological Activation Independent of Hypoxic Conditions

DTx-induced reactive astrocytes have been previously reported to induce microglia activation in the brain [25]. Thus, we hypothesized that DTx-induced reactive astrogliosis will exacerbate the microglia activation caused by hypoxia. To test this hypothesis, we quantified microglia reactivity via changes in morphology from a resting ramified state to a more amoeboid state. Microglia were visualized via IBA-1 (green) immunostaining (Figure 4A). We found that, in PBS vehicle control tissues, hypoxia induced a significant morphological activation of microglia, as quantified by the transformation index (PBS normoxic 100.3 ± 36.59, PBS hypoxic 67.39 ± 33.51 Mann–Whitney *T*-test *p* value < 0.0001). A similar response was observed in response to the DTx treatment (DTx normoxic 64.55 ± 22.54, DTx hypoxic 72.81 ± 34.68, DTx normoxic vs. PBS normoxic Mann–Whitney *T*-test *p* value < 0.0001, DTx hypoxic vs. PBS normoxic Mann–Whitney *T*-test *p* value < 0.0001). However, the combination of DTx and hypoxia did not exacerbate the microglia’s morphological changes (DTx normoxic vs. DTx hypoxic Mann–Whitney *T*-test *p* = 0.2308) (Figure 4C). These data suggest that while both hypoxia and DTx administration induce microglia activation, the combination of DTx and hypoxia does not exacerbate this inflammatory response in terms of changes in microglia morphology.

### 3.5. DTx-Induced Retinal Pathology Is Not Explained by the Expression of Standard Inflammatory and Anti-Inflammatory Mediators

**Figure 5:**
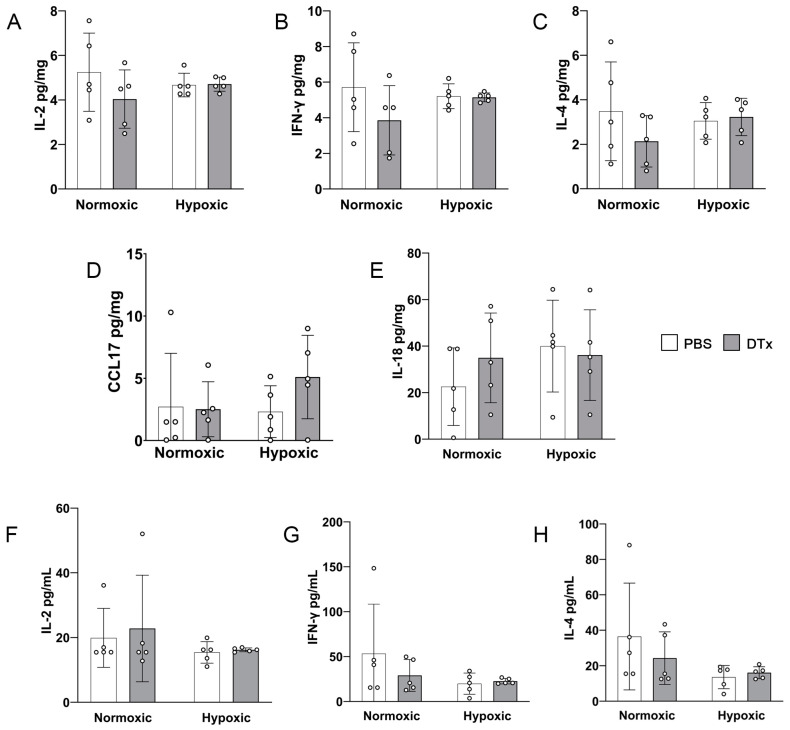
CNS and serum cytokine expression are not affected by hypoxia in the GFAP-Cre^ERT2^:Rosa26^iDTR^ mouse model. (**A**–**E**) Protein expression levels from brain (pg/mg) for IL-2, IFN-γ, IL-4, CCL17, and IL-18, respectively. (**F**–**H**) Protein expression levels from serum (pg/mL) for IL-2, IFN-γ, and IL-4, respectively. Data were analyzed for statistical significance between PBS- and DTx-treated animals under normoxic and hypoxic conditions. Data are presented as mean ± SD, n = 5 mice per group, and dots indicate values for individual animals.

We next sought to test for changes in pro-inflammatory and anti-inflammatory mediators induced by the DTx treatment or hypoxic conditions. We utilized both protein lysate from brain tissue to examine CNS-specific responses and peripheral blood serum to measure systemic responses. We quantified the levels of pro-inflammatory cytokines IL-1β, IL-2, IL-6, IFN-γ, and TNF-α; anti-inflammatory cytokines IL-4, IL-5, IL-10, and CCL-17; the microglia proliferation marker GM-CSF; and the hypoxia inducible factor 1a regulating IL-18 (Table 2) to allow for an assessment of broad inflammatory and anti-inflammatory responses. Serum samples were also used to determine the systemic effects of hypoxia [31]. The brain and serum samples showed similar patterns of expression, with a higher concentration of cytokines detected in the serum. We did not detect changes in pro-inflammatory cytokines in blood or brain samples in response to a DTx or hypoxic treatment (Figure 5 and Table 2). We also found no changes in anti-inflammatory mediators, hypoxic response IL-18, or microglia proliferation marker GM-CSF (Figure 5 and Table 2). These data suggest that neither the DTx treatment nor hypoxia alter the immunomodulatory proteins tested in this study.

## 4. Discussion

**Figure 6:**
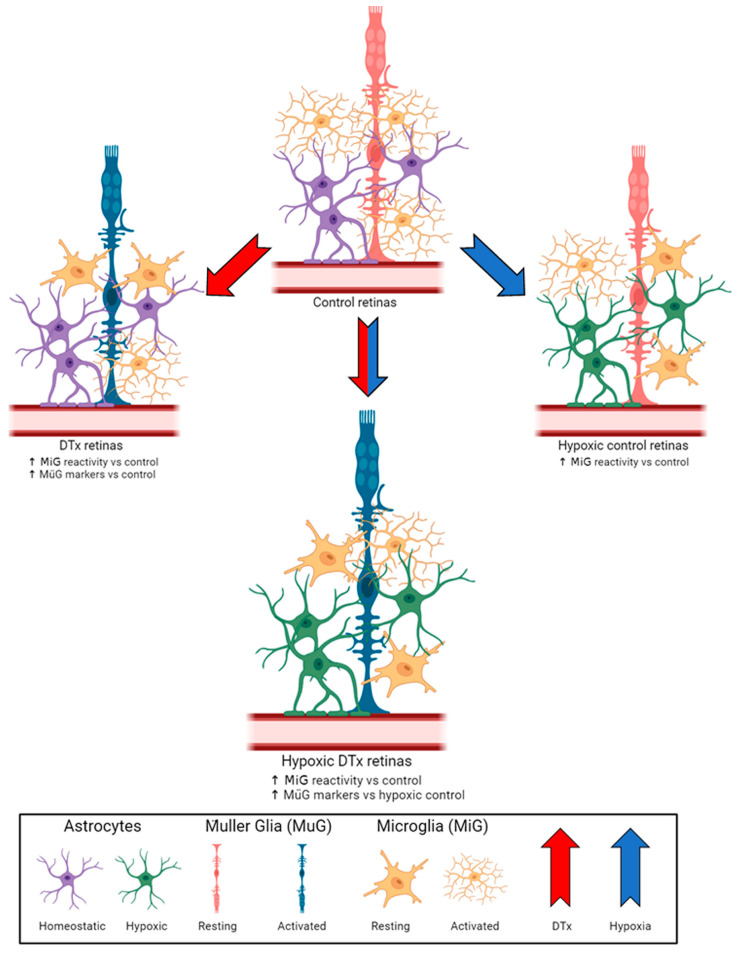
Project summary. Hypoxia does not exacerbate the microglia activation in the GFAP-Cre^ERT2^:Rosa26^iDTR^ mouse model.

As previously reported in brain tissues, the GFAP-Cre^ERT2^:Rosa26^iDTR^ mouse system did not induce an astrocyte depletion in its retinal tissues [25]. We observed evidence of astrocyte hypotrophy in response to hypoxic conditions, with a decrease in GFAP immunoreactivity that was not observed in DTx-treated hypoxic tissues (Figure 6). Interestingly, DTx-treated astrocytes appear to induce Müller gliosis due to a significant increase in their vimentin-immunoreactive area. While Müller glia cells do express GFAP under pathological conditions, they do not transcribe GFAP under homeostatic conditions [32]. The increase in vimentin expression in normoxic TAM- and DTx-treated retinas suggests that this Müller gliosis is in response to DTx-mediated astrocyte responses rather than a response to hypoxic conditions [33].

While OIR is commonly used to investigate the effects of hypoxia on animal systems, there are some drawbacks when attempting to model the hypoxic component of DR. OIR protocols induce hypoxic pathology by placing neonates (P7) in a high-oxygen environment (75% O_2_) for 5 days before being returned to normoxic conditions at P12 [34]. Since vascular development occurs between P7 and P9 [35], this model does not fully recapitulate adult biological processes. DR, especially in type-2 diabetics, is a disease that manifests after the retinal vasculature is fully developed. Therefore, we utilized a model of systemic hypoxia in which adult GFAP-Cre^ERT2^:Rosa26^iDTR^ animals were exposed to conditions of 7.5% O_2_. Most relevant from our results is that the GFAP-Cre^ERT2^:Rosa26^iDTR^ model is not conducive to astrocyte depletion in the retina and that hypoxic astrocytes appear to have a protective role. Therefore, future studies on inducing a switch to a transcriptional signature for anti-inflammatory astrocytes may provide us with ways to regulate retinal pathology in DR.

Similar to other studies [27,36], we observed microglia reactivity via the morphological changes occurring under hypoxia and in DTx-stimulated astrocytes under normoxic conditions. However, in the DTx-treated cohort we did not observe an increase in microglial reactivity in response to hypoxia compared to their PBS-treated hypoxic control. These data suggest that astrocyte reactivity and hypoxia could induce opposing effects on microglia. To further investigate these microglial responses, we examined CNS-specific and systemic cytokine responses, specifically looking for markers of inflammatory activation, IL-6, and anti-inflammatory activation, IL-10 [37]. We did not observe an increase in IL-6 or IL-10 due to the combination of DTx and hypoxic treatments, suggesting neither a net inflammatory nor anti-inflammatory microglia response. Additionally, microglia can be activated by exogenous signals such IFN-γ and HIF-1 α, driving their inflammatory activation or anti-inflammatory activation by IL-4 or HIF-2α [38,39]. We did not observe evidence of a HIF-1α response, with no changes in IL-18, an upstream regulator of HIF-1α, or IL-1β, a downstream response. Nor was there a change in IL-4 expression, an upstream regulator of HIF-2α. This raises the possibility that by day 7 of hypoxia there is an increase in HIF-3α, a negative regulator HIF-1α/HIF-2α that induces increases in RBC production via increased erythropoietin expression [40]. We also did not observe a change in CCL17, a neuroprotective signaling chemokine produced by the neurons that interacts with glial receptor CCR4, expression under any experimental conditions [41]. Our results agree with previously published work, and the lack of cytokine response, while surprising, appears to be a result of the duration of hypoxic conditions. In a study examining the effect of high altitude on cerebral edema, mice were administered a single LPS injection before being exposed to high-altitude conditions (10.16% O_2_) for 6 h, 1 day, or 7 days [42]. The authors reported a transitory increase in pro-inflammatory cytokines IL-1β, IL-6, IL-10, TNF-α, and IFN-γ at the 6 h time point, but these returned to normal by day 7 [42]. The immediate cytokine response suggests that the inflammation is likely mediated by LPS not by hypoxic conditions; results which align with our data. This suggests that reactive astrocytes do not alter the inflammatory profile of the hypoxic retina. In addition, the data do not support reactive astrocytes having a role in altering vascular pathology; with no change in fibrinogen’s intra-vascular or extra-vascular accumulation. Since vascular pathology and leakage was reported in the mouse brain, when using a model of less severe (8% O_2_) hypoxia, by day 7 [9], our data suggest that reactive astrocytes exert a vasculo- and neuro-protective role in hypoxic retinas.

The vascular, microglial, and neuronal pathology seen does not support the alternative hypothesis that DTx-induced reactive astrogliosis exacerbates an inflammatory, hypoxia-induced retinal pathology. Instead, our data reveal a more complex signaling between the astrocytes, Müller glia, and microglia that needs further investigation. This could be further explored by examining the astrocyte responses to hypoxia when the microglia response to inflammation is modulated, in genetic models of FKN or CX3CR1 deficiency. Additionally, the mechanism of astrocyte-mediated Müller glia responses warrants further investigation to discover new potential avenues for therapeutic intervention not only in the context of DR but also other retinal diseases, given the ubiquitous involvement of the Müller glia in retinal pathology [27]. Treatments for DR are limited, focusing on controlling angiogenesis and glucose levels; thus, understanding the contribution of reactive astrocytes to the vascular unit under normal and hypoxic conditions holds promise for potentially identifying new glial signaling pathways that could become new therapeutic targets [43].

## 5. Conclusions

DTx treatment in GFAP-Cre^ERT2^:Rosa26^iDTR^ transgenic mice increased GFAP+ astrogliosis and induced microglial morphological activation without affecting neuronal densities. However, the combination of DTx and hypoxia did not exacerbate microglial activation. Overall, reactive astrogliosis did not alter the inflammatory responses or cause vascular damage in the hypoxic retina.

## Figures and Tables

**Table 1:** Materials and methods—antibodies.

Primary						
Target/Cell		Host		RRID		Dilution
ionized calcium binding adaptor molecule-1 (Iba1)/Microglia		Rabbit		AB_839504		1:4000
neuronal nuclei (NeuN)/RGC		Mouse		AB_2298772		1:4000
glial fibrillary acidic protein (GFAP)/Astrocytes		Rat		AB_2532994		1:4000
platelet endothelial cell adhesion molecule (PECAM-1/CD31)/Endothelial cells		Rat		AB_393571		1:500
fibrinogen		Rabbit		AB_578481		1:2000
Vimentin/Müller glia		Chicken		AB_2216267		1:1000
S100β		Rabbit		AB_956280		1:1000
**Secondary**						
Target		Host		RRID		Dilution
anti-rabbit 488		Donkey		AB_2313584		1:1000
anti-rabbit Cy3		Goat		AB_2338006		1:1000
anti-rat Cy5		Donkey		AB_2340694		1:1000
anti-rat Cy3		Goat		AB_2338394		1:1000
anti-mouse Cy3		Goat		AB_2338709		1:1000
anti-chicken Biotin		Donkey		AB_2340363		1:1000
Streptavidin Cy3		N/A		AB_2337244		1:1000

**Table 2:** Cytokine quantifications for serum and brain protein extracts.

Protein		Values ± SD ^1^				Two-Way ANOVA *p* Value	
		PBS Nor	PBS Hyp	DTx Nor	DTx Hyp	PBS Nor vs. Hyp	DTx Nor vs. Hyp
IL-1β	Brain	5.6 ± 1.67	5.18 ± 0.77	4.58 ± 1.22	5.04 ± 0.13	0.9289	0.9107
	Serum	18.87 ± 3.67	19.44 ± 1.92	18.89 ± 4.47	20.42 ± 1.02	0.991	0.8604
IL-2	Brain	5.25 ± 1.76	4.67 ± 0.53	4.04 ± 1.31	4.71 ± 0.32	0.8532	0.7881
	Serum	19.89 ± 9.09	15.39 ± 3.31	22.79 ± 16.46	16.21 ± 0.59	0.8777	0.7002
IL-6	Brain	4.28 ± 1.67	4.78 ± 0.9	3.57 ± 2.06	4.41 ± 0.88	0.9479	0.8027
	Serum	46.42 ± 60.26	19.8 ± 8.19	20.52 ± 6.66	22.34 ± 4.35	0.5333	0.9997
IFN-γ	Brain	5.72 ± 2.49	5.21 ± 0.7	3.86 ± 1.94	5.15 ± 0.25	0.9593	0.6021
	Serum	53.25 ± 55	19.88 ± 11.8	29.05 ± 17.71	22.65 ± 3.09	0.3149	0.9856
TNF-α	Brain	14.27 ± 6.94	13.17 ± 1.38	9.95 ± 4.64	13.15 ± 1.32	0.9762	0.647
	Serum	87.91 ± 102.76	44.44 ± 17.32	63.75 ± 55.98	48.97 ± 9.74	0.6604	0.9786
IL-4	Brain	3.48 ± 2.22	3.05 ± 0.82	2.13 ± 1.16	3.23 ± 0.84	0.9599	0.6046
	Serum	36.48 ± 30.1	13.59 ± 6.54	24.27 ± 14.83	16.08 ± 3.37	0.193	0.8739
IL-5	Brain	3.52 ± 1.38	2.92 ± 0.33	2.48 ± 1.01	2.97 ± 0.24	0.7045	0.8145
	Serum	18.88 ± 4.84	16.1 ± 5.19	17.64 ± 5.76	16.66 ± 3.43	0.8048	0.9886
IL-10	Brain	13.3 ± 5.71	12.68 ± 1.62	10.1 ± 4	12.32 ± 0.57	0.9924	0.7648
	Serum	86.63 ± 86.11	54.46 ± 24.69	49.28 ± 35.74	57.15 ± 11.76	0.7251	0.9939
CCL-17	Brain	2.71 ± 4.29	2.32 ± 2.08	2.51 ± 2.21	5.1 ± 3.35	0.9971	0.5689
	Serum	42.31 ± 34.18	48.4 ± 30.63	53.75 ± 55.28	83.72 ± 24.76	0.9968	0.7165
GM-CSF	Brain	13.1 ± 4.2	11.99 ± 1.63	10.33 ± 3.53	11.79 ± 0.61	0.9278	0.8535
	Serum	43.33 ± 26.98	50.74 ± 6.91	58.37 ± 57.07	49.5 ± 7.46	0.9826	0.9709
IL-18	Brain	22.56 ± 16.66	40 ± 19.72	34.94 ± 19.27	36.12 ± 19.49	0.4801	0.9996
	Serum	23.17 ± 26.86	16.82 ± 8.5	41.82 ± 50.3	16.98 ± 6.05	0.9915	0.6493

^1^ pg/mg for brain samples; pg/mL for serum.

## Data Availability

The datasets used and/or analyzed during the current study are available from the corresponding author on reasonable request.

## Supplementary Materials

The following supporting information can be downloaded at: https://www.mdpi.com/article/10.3390/biom14050567/s1, Table S1: Statistical *p* values using non-parametric *T*-test.
